# New Approaches with Different Types of Circulating Cathodic Antigen for the Diagnosis of Patients with Low *Schistosoma mansoni* Load

**DOI:** 10.1371/journal.pntd.0002054

**Published:** 2013-02-28

**Authors:** Rafaella Grenfell, Donald A. Harn, Smanla Tundup, Akram Da'dara, Liliane Siqueira, Paulo Marcos Zech Coelho

**Affiliations:** 1 Schistosomiasis Laboratory, Rene Rachou Research Center, Oswaldo Cruz Foundation (Fiocruz), Belo Horizonte, Minas Gerais, Brazil; 2 Department of Infectious Diseases, College of Veterinary Medicine and the Center for Tropical and Emerging Diseases, University of Georgia, Athens, Georgia, United States of America; 3 Tufts University, Grafton, Massachusetts, United States of America; Centers for Disease Control and Prevention, United States of America

## Abstract

**Background:**

Schistosomiasis mansoni is a debilitating and sometimes fatal disease. Accurate diagnosis plays a key role in patient management and infection control. However, currently available parasitological methods are laborious and lack sensitivity. The selection of target antigen candidates has turned out to be a promising tool for the development of more sensitive diagnostic methods. In our previous investigations, the use of crude antigens led to false-positive results. Recently, focus has been given to highly purified *Schistosoma mansoni* antigens, especially to circulating antigens.

**Method:**

Thus, our main goal was to test different types of circulating cathodic antigen glycoprotein (CCA), as “crude antigen,” the protein chain of recombinant CCA and two individual peptides. These schistosome proteins/peptides were tested in a new diagnostic method employing immunomagnetic separation based on the improvement of antigen–antibody binding.

**Principal Findings:**

Use of recombinant CCA as a diagnostic antigen allowed us to develop a diagnostic assay with high sensitivity and specificity with no false-negative results. Interestingly, the “crude antigen” worked as a good marker for control of cure after praziquantel treatment.

**Conclusions/Significance:**

Our new diagnostic method was superior to enzyme-linked immunosorbent assay in diagnosing low endemicity patients.

## Author Summary

Schistosomiasis mansoni is a debilitating and sometimes fatal disease that affects many individuals in Africa and Brazil. Currently available diagnostic methods are not sensitive for patients with low parasite load which leads to underreported cases. The selection of target diagnostic antigen candidates is a promising tool for the development of a new and more sensitive assay. In this study, we focused on different types of circulating cathodic antigen (CCA) for development of an innovative assay. This new assay is called immunomagnetic separation and it uses magnetic microspheres attached to the antigens in order to improve the diagnostic sensitivity. Best results were found when we used the protein chain of the recombinant CCA showing high sensitivity and specificity with no false-negative results. Together, the use of “crude antigen” presented good results for the control of cure. Our new assay was superior to enzyme-linked immunosorbent assay in discriminating positive and negative cases, especially related to patients with low parasite load.

## Introduction

Schistosomiasis is a disease caused by one of six *Schistosoma* species, namely *Schistosoma haematobium*, *S. guineensis*, *S. intercalatum*, *S. mansoni*, *S. japonicum*, and *S. mekongi* [1]. Schistosomiasis occurs in the tropics and subtropics and is among the most important parasitic diseases worldwide, with a considerable socioeconomic impact [2]. Seventy-four countries are endemic, with roughly 120 million individuals being symptomatically infected and 20 million being severely affected [3]. Moreover, schistosomiasis represents an increasing problem in non-endemic areas, due to the growing number of immigrants and tourists [4]–[6]. Herein, diagnosis plays a crucial role in the monitoring of early infection as well as efficacy of treatment. Currently, the ‘gold’ standard remains the detection of *S. mansoni* eggs in stools. The Kato-Katz technique is the most widely used copromicroscopic method in epidemiological surveys [7]. However, because of low and sporadic egg production, the risk of having a large percentage of individuals who remain undiagnosed is considerable. Undiagnosed individuals remain infected and contribute to transmission of the disease [8], [9].

Immunodiagnostic techniques are rapid, sensitive, convenient, and easy to apply, and hence they have been used to estimate infection rates with the goal of improving diagnosis in epidemiological surveys and identifying individuals to target for treatment [10]–[13]. Nonetheless, low specificity is frequently seen in immunodiagnostic assays, largely due to the use of crude antigens that contain many antigens that might be shared with unrelated pathogens. The systematic purification of antigens from *Schistosoma* spp. should allow the development of new anti-schistosome antibodies that might become valuable diagnostic tools [14], [15]. Antigens excreted by adult worms into the circulation of the host, “circulating antigens”, have repeatedly been shown to be potent diagnostic target molecules [16]–[19]. Research on circulating antigens has focused on two genus-specific proteoglycan antigens derived from the schistosome gut: circulating anodic antigen (CAA) and circulating cathodic antigen (CCA). Urine-dipstick diagnostic tests that detect schistosome CCA have the potential to provide more sensitive and rapid detection of for intestinal schistosomiasis in field-based surveys and they showed promising results in Africa [20], although the tests are currently not suitable for rapid mapping of schistosomiasis in areas where *S. mansoni* and *S. haematobium* are co-endemic [21].

For this reason, defined diagnostic antigen(s) that increase sensitivity and specificity of serological assays and that can detect patients with low parasite loads would be of considerable benefit to schistosome control programs. In this regard, a new immunological assay, immunomagnetic separation (IMS), was developed and refined by our group. A benefit of this approach is to effectively concentrate, rather than dilute, patient serum during incubation. We compared IMS to enzyme-linked immunosorbent assay (ELISA) using the same antigens in order to evaluate the effectiveness of this new approach. Therefore we assessed the sensitivity of different forms of CCA for their diagnostic potential. The antigens we focused on were: (i) “crude antigen”, (ii) protein chain of recombinant CCA (CCAr), and (iii) two individual CCA peptides (CCApep1 and CCApep2). A prospective survey was performed with individuals from a low endemicity area for schistosomiasis mansoni. Final analyses were done by comparing IMS results to Kato-Katz and “Three Fecal Test” (TF-Test), as parasitological assays.

Here, we show that a well standardized immunological assay is sensitive and specific for the discrimination of low parasite load cases, by demonstrating that (i) the levels of parasite-specific immunoglobulin G (IgG) are significantly different from positive and negative individuals when IMS is performed with CCAr; (ii) IMS-CCAr achieved the most significant positivity ratio for diagnosis with no false-negative results; and (iii) IMS methodology was superior to ELISA in detecting the presence of schistosome infection in patients with low parasite loads.

## Methods

### Ethics Statement

This project was approved by the Ethical Research Committee of the Rene Rachou Research Center, Oswaldo Cruz Foundation for Animal Use (CEUA L-0023/08) according to the International Guiding Principles for Biomedical Research Involving Animals developed by the Council for International Organizations of Medical Sciences (CIOMS). The Ethical Research Committee of the Rene Rachou Research Center (CEPSH/CPqRR 03/2008) and the National Brazilian Ethical Board (784/2008, CONEP 14886) approved the human study. The study objectives were presented and explained to all participants and written informed consent was obtained through signing a form before admission to this study. Parents/guardians provided written consent on behalf of all child participants.

### Community Survey

A prospective study was performed in the communities of Buriti Seco and Morro Grande in Pedra Preta, a small village in a schistosomiasis-endemic area in the rural region of Montes Claros, Minas Gerais, southeast of Brazil [22]. This area was chosen based on the fact that the population had not been treated for schistosomiasis and also has a low population migration index. Schistosomiasis prevalence rate of 12% was reported in 2005 according to data provided by the Montes Claros Zoonosis Control Centre. The total amount of residents participating in the survey was 201 individuals (93 females and 108 males).

#### Stool sample analysis

Four separate stool samples on four consecutive days were provided for Kato-Katz thick smear examination [7], with a total of 18 slides, prepared as follows for each participant: 12 slides for the first sample and two slides each for the second, third, and fourth samples. Hence, a total of 750 mg of feces was examined per individual (18×41.7 mg). The same samples were analyzed by quantitative TF-Test as previously described [22]. Briefly, samples were passed through a nylon mesh and quantified in metal plates. Each 500 mg portion was transferred to a tube with preservative solution (10% formalin) and processed using ethyl acetate. Samples were centrifuged at 500 *g* for 2 min. The sediment was resuspended in 0.85% saline solution and analyzed using optical microscopy [23].

#### Serum sample processing

Among the 201 individuals, 50 patients with parasite loads varying between 1 and 555 eggs per gram of feces (EPG) were selected to provide serum samples (24 females and 26 males, aged 8–88 years). Individual serum samples were obtained after centrifugation of blood samples at 3,000 *g* for 5 min and kept at −20°C.

#### Treatment of positive cases

Positive individuals were treated with praziquantel in a single dose of 60 mg/kg for children and 50 mg/kg for adults. Infections with other helminths were treated with a single dose of 400 mg albendazole, as recommended by the Brazilian Ministry of Health. Positive patients submitted new stool and sera samples 30 days post-treatment and were retreated.

### Healthy Volunteers

Fifty-three healthy non-endemic area residents (35 females and 18 males, aged 22–65 years) were selected to be used as negative control group. The volunteers presented no medical history of previous schistosomiasis.

#### Confirmatory diagnosis of healthy donors

Confirmatory diagnosis was performed using two ELISA assays for the detection of IgG antibody against soluble worm antigen preparation (ELISA-SWAP) and against soluble egg antigens (ELISA-SEA). Patients reactive for both ELISA assays were removed from the “healthy” cohort.

### CCA Preparation

#### “Crude antigen” containing CCA glycoprotein

Adult worms from *S. mansoni* (LE strain) were obtained by perfusion of hepatic portal system of Swiss female mice (4–6 weeks) 45 days post-infection with 100 cercariae [24]. Adult worms were washed with 0.15 M phosphate buffer saline pH 7.2, submitted to mechanical grinding (Virtiz Precisa; Dietikon, Switzerland) and ultracentrifuged at 25,000 *g* for 1 h at 4°C (Sorvall; Buckinghamshire, United Kingdom). Supernatant was heated to 100°C for 30 min, as previously described [25] then filtered through a 50 kDA exclusion filter (Millipore Amicon, Sigma-Aldrich; St. Louis, United States of America). Final purified product was dialyzed against saline solution 0.9% for 48 h at 4°C and maintained at −20°C prior to use, after protein concentration analysis (Nanodrop, Thermo Scientific 2000; Rockford, United States of America). A silver stained Tris-glycine SDS-PAGE (12% gel) was performed to assess purity [26].

#### CCAr

Adult worms were homogenized by glass homogenizer with 1 ml of Trizol (Invitrogen; Grand Island, United States of America), incubated for 10 min at 25°C when 200 µl of chloroform was added. This suspension was centrifuged at 15,000 *g* for 15 min at 4°C and the upper layer was reserved. RNA was precipitated by addition of 500 µl of isopropanol, incubated for 10 min and centrifuged at 4°C for 10 min. Cold 75% ethanol was added to the pellet followed by a new centrifugation step. Ethanol was removed and the final pellet dried, ressuspended in RNase-free water and kept at −20°C until use. cDNA was obtained according to the manufacturer's instructions (SuperScript II Reverse Transcriptase, Invitrogen; Grand Island, United States of America).

CCA gene reference sequence was obtained from the database of the National Center for Biotechnology Information (NCBI) [27], GenBank AAB53003.1. To express the CCA domain, gene-specific primers were designed as follows: sense 5′- CCC*GGATCC*ATGACGTTTGATTTCATGTTAAAG - 3′ and antisense 5′- GGG*CTCGAG*TAGGGAGTTAATCATTTGATTCATAGC - 3′ which contain *Bam*HI and *XHO*I restriction sites (italicized), respectively. The PCR conditions through 32 cycles were 95°C for 45 sec as denaturing step, 60°C for 45 sec as an annealing step and 72°C for 1 min as an elongation step. The PCR product was first subcloned into the pCR-Blunt II TOPO plasmid (Invitrogen; Grand Island, United States of America) and transformed into TOP10 competent cells. Recombinant plasmid DNA was isolated and digested with the restriction enzymes. The resulting fragment was purified and subcloned into *Bam*HI-*Xho*I-cleaved pET21a. The final recombinant expression plasmid (CCA-pET21a) was sequenced and subsequently introduced into *Escherichia coli* strain BL21 Gold competent cell (Agilent Technologies; La Jolla, United States of America). Cells were grown overnight at 37°C in Luria Bertani (LB)-medium containing 75 µg/ml ampicillin. The overnight culture was diluted 100-fold in LB-medium and grown until an optical density (OD) (600 nm) for 0.5 was reached. To induce protein expression, isopropyl β-D-thiogalactoside (IPTG) 1.0 mM was added and the cells were grown for another 3 h. Cells were harvested by centrifugation and sonicated. Purification was done by affinity chromatography on His-Trap columns (Amersham/Pharmacia; Pittsburgh, United States of America). The homogeneity of the recombinant protein was analyzed by silver stained Tris-glycine SDS-PAGE (12% gel) [26].

#### CCA peptides

CCA sequence was retrieved from NCBI/protein and the full length protein sequence was subjected to B cell prediction at BCPreds: B-cell epitope prediction server 1.0 [28]–[30]. Two best conformations of surface exposed B cell epitope sequences having the cut-off value for BCPreds (>0.9) were taken into consideration. Peptides (Table 1) were synthesized (Mimotopes; San Diego, United States of America).

**Table 1:** Predicted B cell epitopes for *Schistosoma mansoni* CCA.

Reference no.	Peptide sequence	Amino acid positions	BCPred score
1	Pro-Asn-Pro-Ser-Asp-Asp-Ser-Ser-Asn-Ser-Gly-Thr-Ile-Ser-Gly-Asn-His-Ser-Asp-Glu	307	1
2	Lys-Gln-Leu-Glu-Gln-Leu-Lys-Ile-Glu-Asn-Lys-Thr-Leu-Arg-Asn-Ser-Leu-Asp-Glu-His	83	0.93

#### Purification evidence for CCA

A confirmation method was used for each CCA antigen obtained. Microtiter plates MaxiSorp Surface (NUNC, Thermo Scientific; Roskilde, Denmark) were coated with 100 µl/well of 1 µg/ml of each antigen (“crude antigen”, CCAr and each individual CCA peptide) diluted in 0.05 M carbonate-bicarbonate buffer pH 9.6 for 16 h at 4°C. As positive controls, we included SWAP. Plates were washed three times with 0.15 M phosphate buffered saline pH 7.2 with 0.05% of Tween 20 (LGC Biotecnologia; São Paulo, Brazil) (washing buffer), then blocked by incubation with 2.5% skim milk at 37°C for 1 h. Then, 100 µl/well of a peroxidase conjugated IgG1 monoclonal antibody against CCA was added (1∶8,000) (lot 5F4.B4, University of Georgia, Monoclonal Antibody Facility; Athens, United States of America) then incubated at RT for 1 h. Plates were washed in PBS-T 0.05%, then 100 µl of substrate 3,3′,5,5-tetramethylbenzidine solution (Invitrogen; Grand Island, United States of America) were added and the reaction stopped after 15 min of incubation by addition of 50 µl/well of 2 N sulfuric acid. Results were obtained as OD at 450 nm using a microplate reader (Model 3550, Bio-Rad Laboratories; Tokyo, Japan).

### IMS with CCA

Paramagnetic microspheres (0.4 µm) (Estapor Microspheres, Merck; Lyon, France) were sensitized with CCA (10^6^ microspheres with 1 µg/ml of antigen/assay): (i) with “crude antigen”; (ii) CCAr; (iii) CCA pep1; and (iv) CCA pep2. All incubation steps were performed under rotation to improve antigen-antibody binding. For sensitization step, antigens were diluted in 0.05 M carbonate-bicarbonate buffer pH 9.6 for 16 h at 4°C. Microspheres were washed four times with washing buffer using a 1.5 ml tube magnetic base (Invitrogen; Grand Island, United States of America). Non specific-binding was blocked using 20% skim milk proteins in washing buffer at 4°C for 16 h. Microspheres were washed and maintained at 4°C. Prior to use, microspheres were washed, then 100 µl of a non-diluted serum sample were added in duplicate, followed by incubation at 37°C for 1 h. Microspheres were then incubated at 37°C for 1 h with 100 µl of peroxidase conjugated anti-human IgG Fc specific (Sigma-Aldrich; St. Louis, United States of America) diluted 1∶60,000 in washing buffer. Tubes were washed and 100 µl of substrate 3,3′,5,5-tetramethylbenzidine solution (TMB/H_2_O_2_) (Invitrogen; Grand Island, United States of America) were added to each well and the reaction was stopped after 10 min. Using the magnetic base, supernatant were transferred to a microtiter plate and results were obtained as OD at 450 nm in a microplate reader (Model 3550, Bio-Rad Laboratories; Tokyo, Japan).

### Indirect ELISA with CCA

ELISA were standardized based on a technique described elsewhere [31] after some modifications. Microtiter plates MaxiSorp Surface (NUNC, Thermo Scientific; Roskilde, Denmark) were coated with 100 µl per well of CCA diluted at 1 µl/ml in 0.05 M carbonate-bicarbonate buffer pH 9.6 for 16 h at 4°C. Plates were washed three times, then blocked by addition of 2.5% skim milk and incubating at 37°C for 1 h. Plates were washed, then 100 µl of individual serum sample diluted 1∶100 in 0.15 M phosphate buffer saline pH 7.2 were added in duplicate followed by incubation for 1 h. Plates were then washed and incubated with peroxidase conjugated anti-human IgG Fc specific (Sigma-Aldrich; St. Louis, United States of America) diluted in washing buffer at 1∶60,000. 100 µl of substrate solution were added to each well and the reaction was stopped after 10 min of incubation and OD at 450 nm determined by microplate reader. The cut-off value of each ELISA method was determined by receiver operating curve (ROC) and they were defined as 0.250 for ELISA-crude Ag, 0.103 for ELISA-CCAr, 0.117 for ELISA-CCApep1 and, 0.166 for ELISA-CCApep2 (A = 0.765, 0.924, 0.954, 0.824, respectively). Positive and negative controls were assayed for both techniques.

### Statistical Analysis

Data derived from absorbance values were analyzed with Minitab software (Minitab Inc, United States of America) by Kolmogorov-Smirnov normality test. Normal distributed data were analyzed by Student's *t* test and non-normal distributed data were analyzed by Mann-Whitney test. Comparisons between methods were done by chi-square (χ^2^) analysis (p<0.05 as significance level). The sensitivity, specificity, cut-off values, and likelihood ratios were determined with Prism 4.0 software. Agreement between methods was measured using the Cohen coefficient [32] and analyzed according the Landis & Koch definition [33], with software ComKappa 2.0: 1.00-0.81 almost perfect agreement; 0.80-0.61 substantial agreement; 0.60-0.41 moderate agreement; 0.40-0.21 fair agreement; 0.20-0 slight agreement; <0 poor agreement.

### Accession Numbers

CCA gene reference sequence was obtained from the database of the National Center for Biotechnology Information (NCBI), GenBank AAB53003.1.

## Results

### CCA Preparation

**Figure 1:**
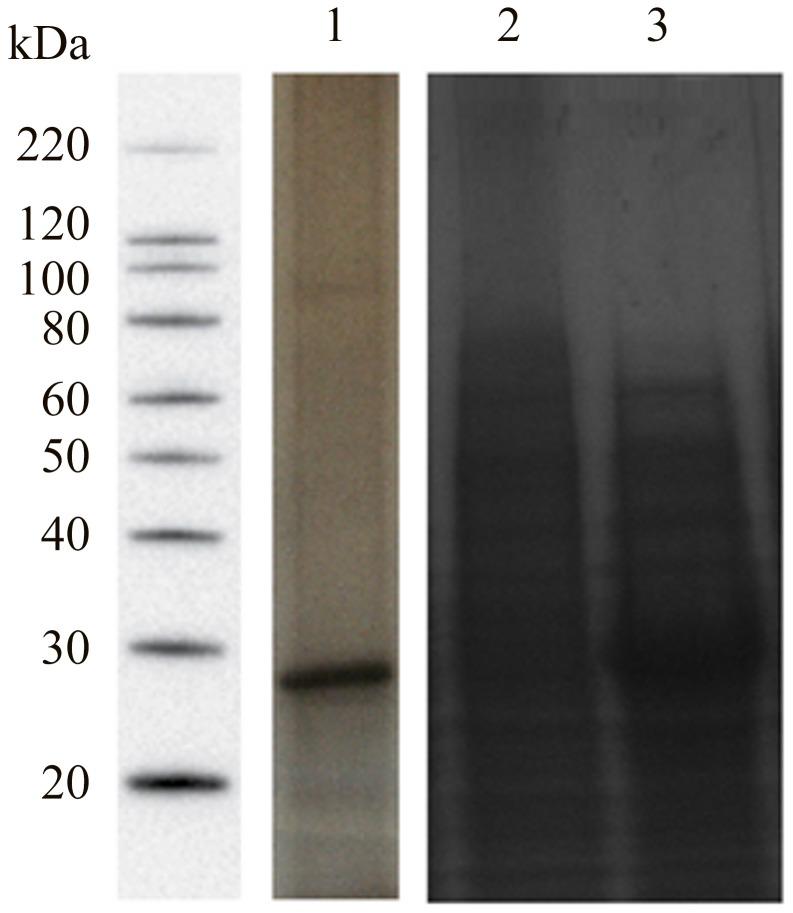
SDS-PAGE analysis of the “crude antigen” and the protein chain of recombinant CCA. Aliquots of samples corresponding to the final product of adult worm extract submitted to purification steps (2) and, the CCA recombinant protein expressed in *E. coli* before (3) and after (4) induction with IPTG were subjected to silver stained SDS-PAGE analysis. Electrophoresis was done using 12% Tris-glycine gels. Molecular weight standards are shown in (1).

Four different forms of CCA were obtained and tested in IMS assays. Initially, the “crude antigen” was obtained from adult worm extracts and the CCAr were induced in *E. coli*. Final products are shown on Figure 1 and both demonstrated a 30 kDa protein, correlating with previously reported characteristics of CCA [25].

**Figure 2:**
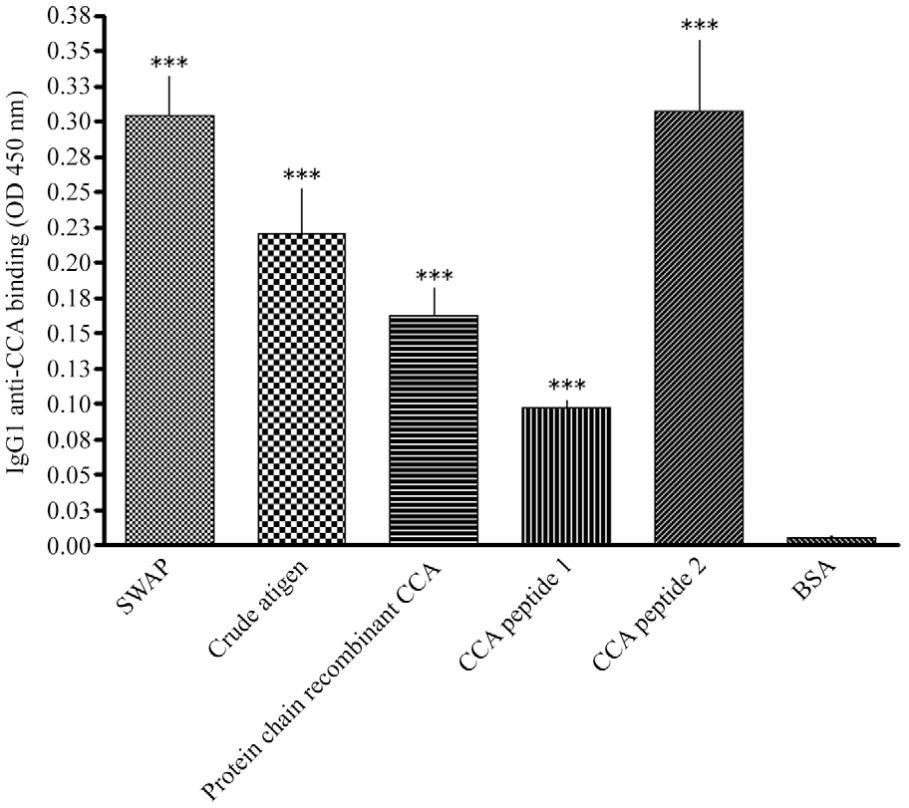
Binding of CCA-specific monoclonal IgG1 antibody to the four CCA antigens. Antigens represented by bars are: SWAP – soluble worm antigen preparation, as the positive control; “crude antigen”; protein chain of recombinant CCA; CCA peptides 1 (BCPred Score = 1) and 2 (BCPred Score = 0.926). Each OD value is representative for the mean of four absorbance values. Statistical differences for comparisons done to BSA are represented by *** (*p*<0.05) using Student's *t* test. Artwork created by Prism 5.0 software.

Afterwards, two CCA peptides were synthesized based on predicted B cell epitopes. These four CCA were then tested as diagnostic assay candidates, using a monoclonal IgG1 antibody against *S. mansoni* CCA (lot 5F4.B4, University of Georgia, Monoclonal Antibody Facility, Athens, United States of America). Results are shown on Figure 2 where a significant reaction was seen for all four CCA in comparison to bovine serum albumin (BSA) as our negative control.

### IMS Validation for Low Endemicity Area Residents

The prospective study involved the communities of Buriti Seco and Morro Grande in Pedra Preta, southeast Brazil. These communities are areas of low endemicity for schistosomiasis mansoni, and with low migration index and no history of previous treatment. Among the 201 individuals participating in the survey, 50 patients including adults and children were selected to provide serum samples (24 females and 26 males). These patients were first diagnosed by Kato-Katz and TF-Test and results showed a parasite load range between 1 and 555 EPG among the group. All patients were treated as recommended and they resubmitted stools for Kato-Katz testing 30 days post treatment when serum samples were obtained. Retreatment was done in all reinfection cases.

**Figure 3:**
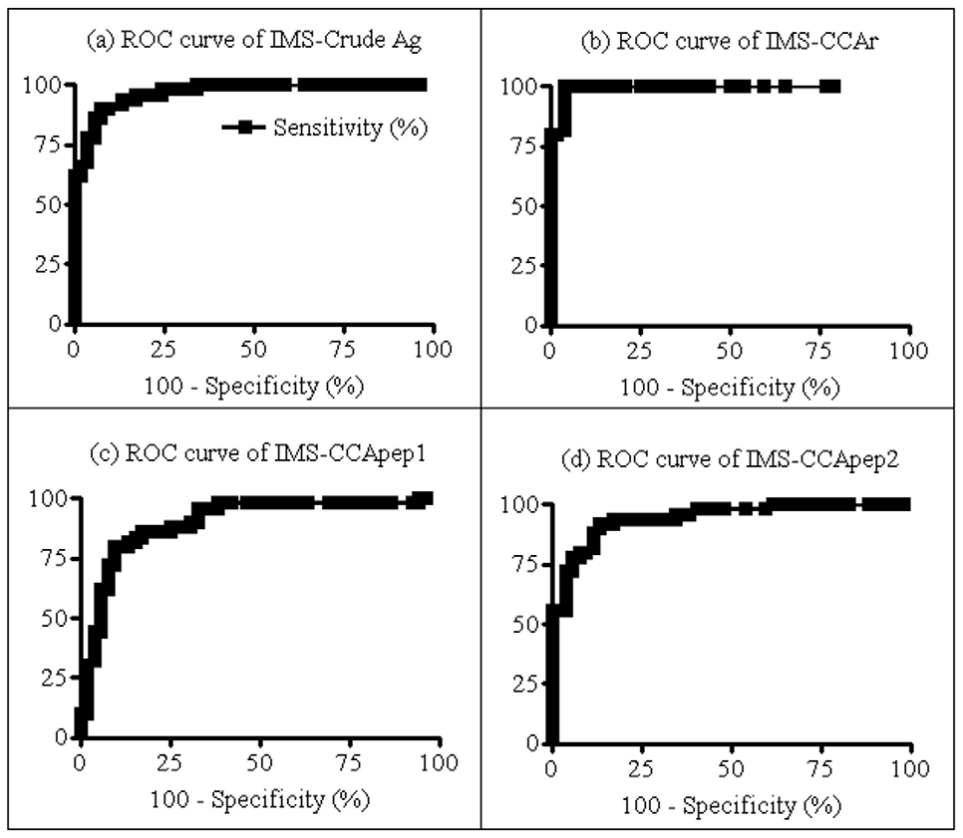
ROC curves of each IMS protocol. In (a) IMS-crude Ag (A = 0.97, *p*<0.05); (b) IMS-CCAr (A = 0.99, *p*<0.05); (c) IMS-CCApep1 (A = 0.90, *p*<0.05); and (d) IMS-CCApep2 (A = 0.94, *p*<0.05). Artwork created by Prism 5.0 software.

The 50 serum samples selected from people of Pedra Preta, together with the healthy donors' serum samples were screened by IMS using the four different antigens described: sensitized with “crude antigen” (IMS-crude Ag), CCAr (IMS-CCAr), CCA pep1 and CCApep2 (IMS-CCApep1 and IMS-CCApep2). The 53 healthy donors were initially tested for antibodies to schistosomes by ELISA-SWAP and ELISA-SEA. Only one individual was reactive for both antigens and was not therefore removed from the healthy (negative) control group. Further, cut-off value, positivity ratio, sensitivity, and specificity of each IMS methodology were determined by ROC, which are represented in Figure 3.

**Figure 4:**
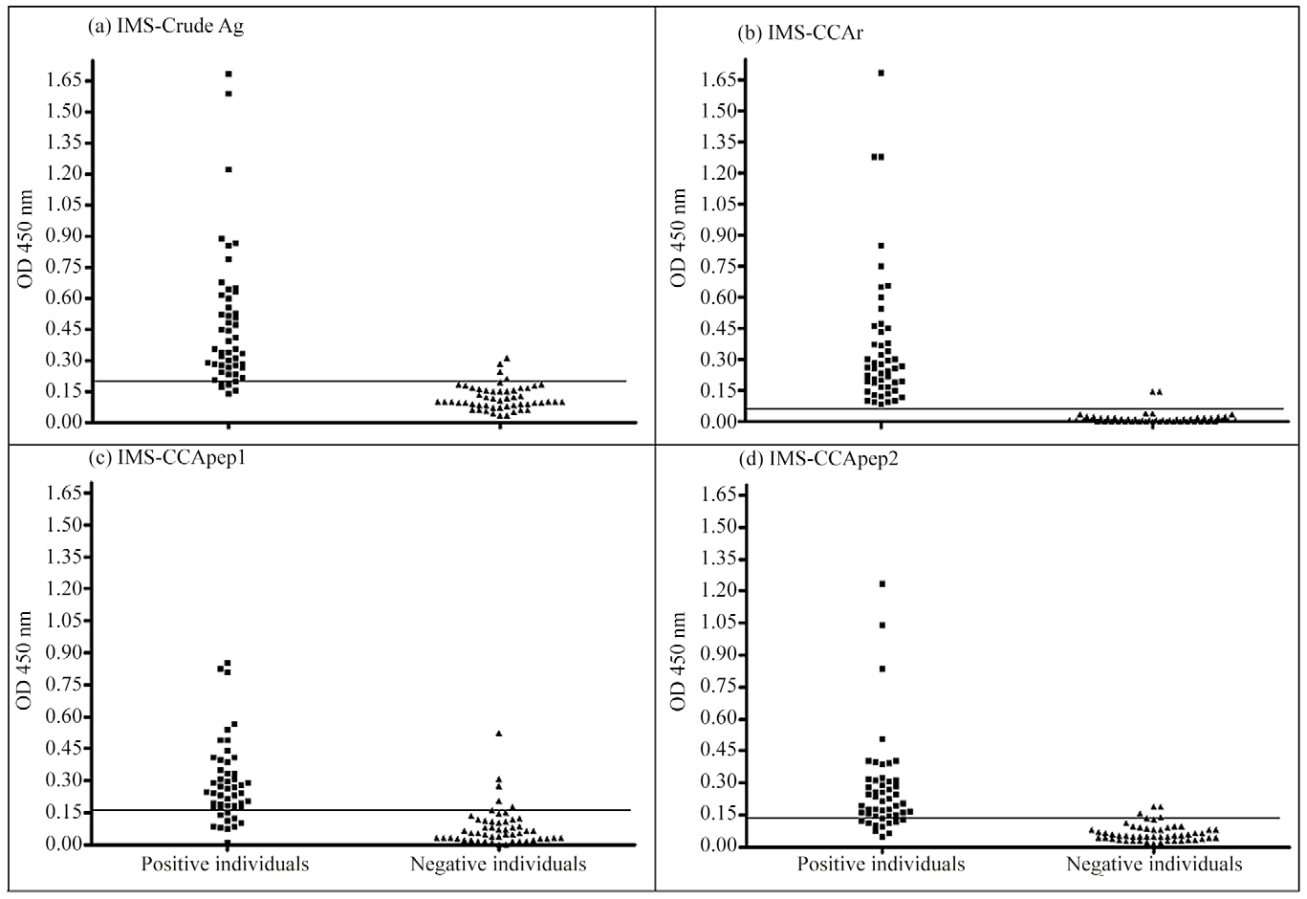
Individual analysis of 102 serum samples by the IMS protocols. Each OD value is representative for the mean of four absorbance values. Cut-off values are represented by bars. In (a) IMS-crude Ag (cut-off = 0.20); (b) IMS-CCAr (cut-off = 0.06); (c) IMS-CCApep1 (cut-off = 0.16); and (d) IMS-CCApep2 (cut-off = 0.13). Artwork created by Prism 5.0 software.

IMS-crude Ag presented a sensitivity of 90% and a specificity of 92% for a cut-off value of 0.197, which showed that the “crude antigen” might be considered a good marker for schistosome infection with five missing positive patients and four missing negative individuals. Moreover, IMS using CCAr showed an excellent result providing a sensitivity of 100% and specificity of 96% for a cut-off of 0.063, where only two negative individuals presented false-positive results. Finally, IMS using CCA peptides showed similar effectiveness with the same sensitivity (80%) and a specificity of 90% and 92%, respectively for cut-off values of 0.164 and 0.133. When analyzing false-positive and false-negative results, we could see that the use of these 20 amino acids peptides decreased the diagnostic effectiveness with 10 false-negative results for both peptides, five for IMS-CCApep1, and four for IMS-CCApep2. The positivity ratios achieved by each IMS method were 91% (93/102), 98% (100/102), 85% (87/102) and 86% (88/102), for IMS-crude Ag, IMS-CCAr, IMS-CCApep1 and IMS-CCApep2, respectively. The positivity ratio achieved by IMS-CCAr was significantly higher than the other three IMS assays (χ^2^ = 0.74, *p*<0.05). Figure 4 shows the individual OD for each positive and negative patient as determined by each IMS protocol.

**Figure 5:**
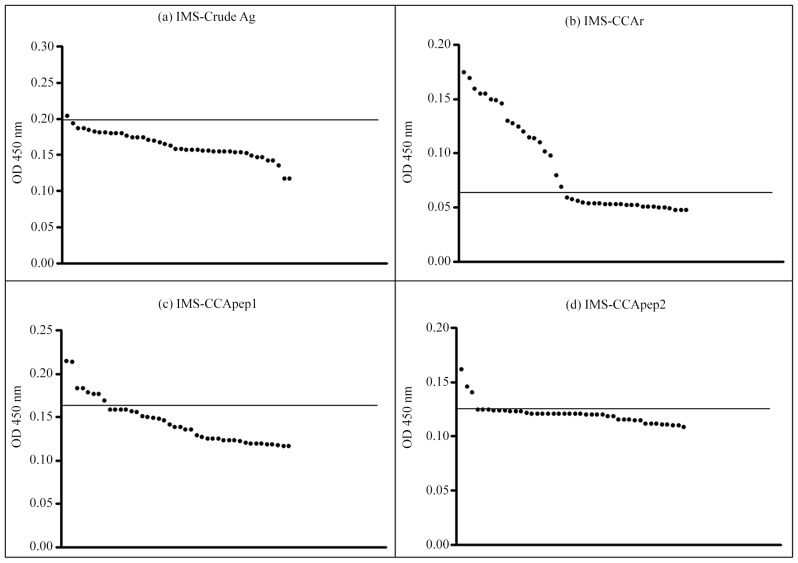
Individual analysis of IgG titer in 30 days after praziquantel administration by IMS protocols. Each OD value is representative for the mean of four absorbance values. Cut-off values are represented by bars. In: (a) IMS-crude Ag; (b) IMS-CCAr; (c) IMS-CCApep1; and (d) IMS-CCApep2. Artwork created by Prism 5.0 software.

Not all the infected patients showed an adequate post treatment follow-up, since no eggs were found in any patient stools 30 days after drug administration. Forty-two of the 50 praziquantel-treated patients agreed to donate serum samples once more. Diagnostic results obtained by the four IMS protocols from both time points were compared with the purpose of detecting any differences in IgG antibody titers. From the observations in each period, 98% of the patients became negative via IMS-crude Ag (41/42), whereas 81% became negative via IMS-CCApep1 (34/42) and 93% via IMS-CCApep2 (39/42). IMS using CCAr identified 55% of the patients as negative for *S. mansoni* 30 days after treatment (23/42), as shown in Figure 5.

### Comparative Analysis of the Effectiveness of IMS with ELISA

In addition to the fact that IMS was standardized with non-diluted serum, the incubation steps were performed under rotation with the purpose of improving antigen-antibody binding and thus, diagnostic sensitivity. To test this hypothesis, “crude antigen”, CCAr, and CCA peptides were used in ELISA (ELISA-crude Ag, ELISA-CCAr, ELISA-CCApep1, and ELISA-CCApep2) and the results were compared to data obtained using IMS analysis. Significant differences were observed in the positivity ratios. Forty-five positive patients were correctly diagnosed by IMS-crude Ag, but only 35 were diagnosed by ELISA-crude Ag (χ^2^ = 0.21, *p*<0.05). All the patients were positive for IMS-CCAr in comparison to 48 patients diagnosed by ELISA-CCAr (χ^2^ = 0.48, *p*<0.05). On the other hand, IMS-CCApep1 and ELISA-CCApep1 presented no difference with 40 positive patients. However, comparing CCApep2, statistical differences were detected with 40 patients diagnosed by IMS-CCApep2 and 37 by ELISA-CCApep2 (χ^2^ = 0.21, *p*<0.05). Analysis of Cohen's kappa index showed a moderate agreement of 0.47 (±0.10) (69/102) between IMS-crude Ag and ELISA-crude Ag. The same agreement was found for IMS-CCApep2 and ELISA-CCApep2 that showed an agreement of 0.48 (±0.11) (66/102). A better agreement was found for IMS-CCAr *versus* ELISA-CCAr and, IMS-CCApep1 *versus* ELISA-CCApep1, which indicated a substantial agreement of 0.66 (±0.10) with a positivity of 84/102 and 0.70 (±0.11) with 75/102, respectively.

Data obtained from a prospective parasitological diagnosis with 18 slides of Kato-Katz plus TF-Test confirmed the low parasite load of residents of Pedra Preta that were infected by *S. mansoni* (1 to 555 EPG). Based on the World Health Organization thresholds [34], most of the infected individuals (48/50) presented low parasite load (1–99 EPG). Hence, those individuals were subdivided into three groups: 1–10 EPG, 11–30 EPG, and >30 EPG in order to determine the sensitivity of each methodology used. Groups were examined by IMS and ELISA methods. Results are shown in Table 2.

**Table 2:** Relation between the number of eggs and IMS and ELISA diagnosis.

Number of eggs per gram of feces (EPG)	n	Positive results detected by each method							
		IMS-crude Ag	IMS-CCAr	IMS-CCApep1	IMS-CCApep2	ELISA-crude Ag	ELISA-CCAr	ELISA-CCApep1	ELISA-CCApep2
1–10	30	26	30	23	24	18	29	24	19
11–30	11	11	11	11	8	8	10	8	9
>30	9	8	9	6	8	9	9	8	9

## Discussion

Population and treatment-based control programs have been successful in reducing the intensity of infection and severe morbidities associated with schistosomiasis. However, transmission remains active in highly endemic areas, and recurring low-level reinfection is likely to be associated with subtle but persistent morbidities [35]–[37]. Adequate case-finding is essential for the effective execution of control programs. Diagnosis has mainly depended upon finding eggs in patients' fecal samples. However, fluctuation in egg output and the chance of missing light infections necessitate repeated examinations [9]. Serologic testing has been used to enhance our ability to detect the disease in order to be more sensitive in demonstrating light infections [10]–[12], [38]. This study applied a multievaluation approach, combining specific antibody detection for four different antigens with an investigation performed on parasitological data in efforts to produce a more field-applicable assay format.

The identification and description of CCA as a constitutional glycoprotein from adult worms gut [39] has allowed the development of assays for detecting antibodies or circulating antigens in urine and serum samples of infected individuals [18], [19], [40]–[42]. When CCA was used in those assays, sensitivity was lower than expected which was partly explained as a consequence of low levels of circulating antigens being regurgitated by adult worms [43], especially in patients with low parasite loads. To solve this problem, we standardized a method called IMS that uses paramagnetic beads in contact with non-diluted serum and is based on incubation steps performed under rotation, allowing an increased antigen-antibody binding. The IMS method was evaluated with four different CCA, including the “crude antigen”, the protein chain of CCAr and, also, two individual peptides of 20 amino acids. Since schistosomiasis epidemiological profiles show an increase in the number of low endemicity areas, the sensitivity of each IMS was validated with patients' samples from an endemic area in southeast Brazil, where most of them showed low parasite load based on 18 Kato-Katz thick smears plus TF-Test.

Although “crude antigen” prepared from *S. mansoni* adult worm showed good results in IMS-crude Ag methodology, CCAr presented more significant results, especially when all the positive cases were properly detected with a sensitivity of 100% and, only two false-positive results, giving rise to a specificity of 96% (χ^2^ = 0.74, *p*<0.05). Whereas IMS-crude Ag achieved 90% sensitivity and 92% specificity, with five false-negative and four false-positive results.

Comparison between the positivity ratios revealed that IMS-CCAr was significantly superior to IMS-crude Ag for diagnosing low endemicity patients (χ^2^ = 0.74, *p*<0.05). The prior structural difference between “crude antigen” and CCAr that justifies their specificity lays on the fact that CCA is present in “crude antigen” sample in its native form as a whole glycoprotein, while the recombinant CCA was expressed in *E. coli* and contains only the protein chain of the native CCA, which was not glycosylated. Native CCA glycoprotein contains 0-linked poly (Le^x^) carbohydrate chains with approximately 25 repeating units. Carbohydrate chains containing multiple Le^x^ determinants have been identified on several glycolipids not only from schistosomes but also from other parasites [44], [45], from human adenocarcinomas [46] and also, circulating granulocytes carry relatively high abundance of branched N-linked polysaccharides having Le^x^ repeating units [47], [48]. Additionally, Le^x^ sequence is particularly immunogenic, playing an important role during inflammatory processes, especially in granulocyte and monocyte adhesion processes and recruiting granulocytes to sites of inflammation [49]. It is conceivable that the use of the native CCA glycoprotein in schistosome diagnosis leads to false-positive results, when IgG antibodies against its most immunogenic portion (the Le^x^ units) can be mistakenly detected. In contrast, the CCA protein sequence of 347 amino acids, obtained by recombinant expression, is exclusively found in the genus *Schistosoma* with no description in any other parasite or human proteins, confirmed by Blast search.

The use of synthetic peptides corresponding to a single continuous epitope may increase the specificity of an immunoassay in the same way that monoclonal antibodies recognizing a single epitope do compared to polyclonal antiserum. Thus prediction of B cell epitopes was performed and the two best conformations were considered. Same identity analysis was done and both peptides were recognized by the CCA-specific monoclonal antibody. When evaluating each peptide for the diagnosis of *S. mansoni* using IMS methodology, data showed similar results for these two methods, as demonstrated by ROC. IMS-CCApep1 presented 80% of sensitivity and 90% of specificity with 10 missing positive cases and five missing negative individuals. IMS-CCApep2 showed the same sensitivity and four missing negative cases, leading to a specificity of 80%. Despite the possible advantage of increasing diagnosis specificity with individual peptides, our data did not show any disparity in specificity between IMS-crude Ag and IMS-CCApep1 or IMS-CCApep2 when similar identification of false-positive cases was found by the three methods.

In a final comparison of the four antigens, CCAr continued to yield a higher positivity ratio of 98% compared to “crude antigen” (91%), CCApep1 (85%), and CCApep2 (86%) (χ^2^ = 0.74, *p*<0.05). Recombinant protein-based diagnosis offers important advantages because higher antigen concentrations can be used, and nonspecific moieties are not present in those proteins, as they may be in crude antigens or in native proteins. Nevertheless, due to the restricted amino acids sequence of a single peptide, the use of each sequence has been deemed impractical. This suggests that for peptides to be used a large pool of epitopes would be required to achieve wide population coverage and the cost would increase significantly.

All the individuals who presented eggs in stools were treated, as recommended by the Brazilian Ministry of Health. These positive patients were invited to resubmit stool samples subjected to the Kato-Katz technique after 30 days of chemotherapy and none of them presented eggs in stool at that time. Forty-two patients were followed up by IMS methodology. A decrease was detected on IgG antibody level for some individuals in the four IMS assays. Forty-one patients presented low IgG levels for IMS-crude Ag, whereas only 23 presented for IMS-CCAr, 34 for IMSCCApep1, and 39 for IMS-CCApep2. Although the dynamics of the post-treatment antibody levels are variable [50], IMS-crude Ag, IMS-CCApep1, and IMS-CCApep2 results were considered extreme. The analysis of the control of cure of IMS-CCAr would benefit from patients' diagnosis in different time points for at least one year. The establishment of antibody titers timeline may help to distinguish the treatments that result in parasitological cure from those that are only partially successful.

The distinctive patient who was positive for IMS-crude Ag was also positive for the other IMS methodologies showing no inconsistency on the diagnosis performed for IMS. The possibility that this patient may have been reinfected or presented immature worms at the moment of treatment cannot be excluded. No data involving the detection of CCA in serum of patients infected for less than 6 weeks have been published to date. However, CCA can be detected in mice 3 weeks post-infection, plus freshly transformed schistosomula, or isolated adult worms excrete CCA *in vitro* immediately after transformation [51].

Since other investigators have reported low sensitivity values for the detection of antibodies against CCA or the circulating antigen itself in urine and serum samples using immunological assays for *S. mansoni* diagnosis [19], [41], [42], [52], we compared our IMS data with results obtained by ELISAs that were standardized with the same CCA antigens. The main differences of these two methodologies were: (i) IMS was performed with non-diluted serum, while ELISA used only diluted samples (1∶100), and (ii) incubation steps were done under rotation for IMS, different from the ELISA. As expected, the use of “crude antigen”, CCAr, and CCApep2 on IMS methodology showed significant higher sensitivity than ELISAs. Indeed, IMS-crude Ag were capable of detecting 10 extra positive patients than ELISA-crude Ag (χ^2^ = 0.21, *p*<0.05), whereas IMS-CCAr and IMS-CCApep2 detected two and three extras positive patients, respectively than ELISAs (χ^2^ = 0.48 and 0.21, *p*<0.05). The superiority of IMS in detecting positive cases was evident when low egg burden patients were divided into three groups based on parasite load. Interestingly, our data showed that IMS-crude Ag, IMS-CCAr and IMS-CCApep2 continued to show a higher sensitivity than ELISA even for patients with parasite loads as low as 1–10 EPG, and this observation was especially demonstrated by IMS-CCAr. Cohen's kappa index confirmed a moderate agreement between IMS and ELISA for CCA “crude antigen” (0.47±0.10) and CCApep2 (0.48±0.11), and a substantial agreement for CCAr (0.66±0.10) and CCApep1 (0.70±0.11).

The present study was undertaken to develop an assay that might be more field applicable than the ELISA for testing of individuals from low endemicity areas. The comparison between different types of CCA allowed the evaluation of the specific capability of each assay in diagnosing positive and negative individuals and the occurrence of false-positive and/or false-negative results. IMS-CCAr presented the most significant positivity ratio for the primary diagnosis. Due to the restricted single epitopes of individual peptides, the 20 amino acids sequence used here showed no advantages in comparison to other antigens. Results revealed that the detection of specific IgG antibody against CCA antigens in serum may be used as an important tool for the diagnosis of *S. mansoni* for patients with low parasite load.

## Supporting Information

Checklist S1 STARD Checklist. STARD checklist for reporting the study of diagnostic accuracy.
(PDF)

Checklist S2 Flowchart. Diagram that represents the sequencing of operations for the prospective study performed in the communities of Buriti Seco and Morro Grande in Pedra Preta, Brazil.
(PDF)
