# Supplementary figures and images for: New Approaches with Different Types of Circulating Cathodic Antigen for the Diagnosis of Patients with Low Schistosoma mansoni Load
Source: PLoS Negl Trop Dis. 2013 Feb 28;7(2):e2054. doi: 10.1371/journal.pntd.0002054 (PMC3585039; doi:10.1371/journal.pntd.0002054)

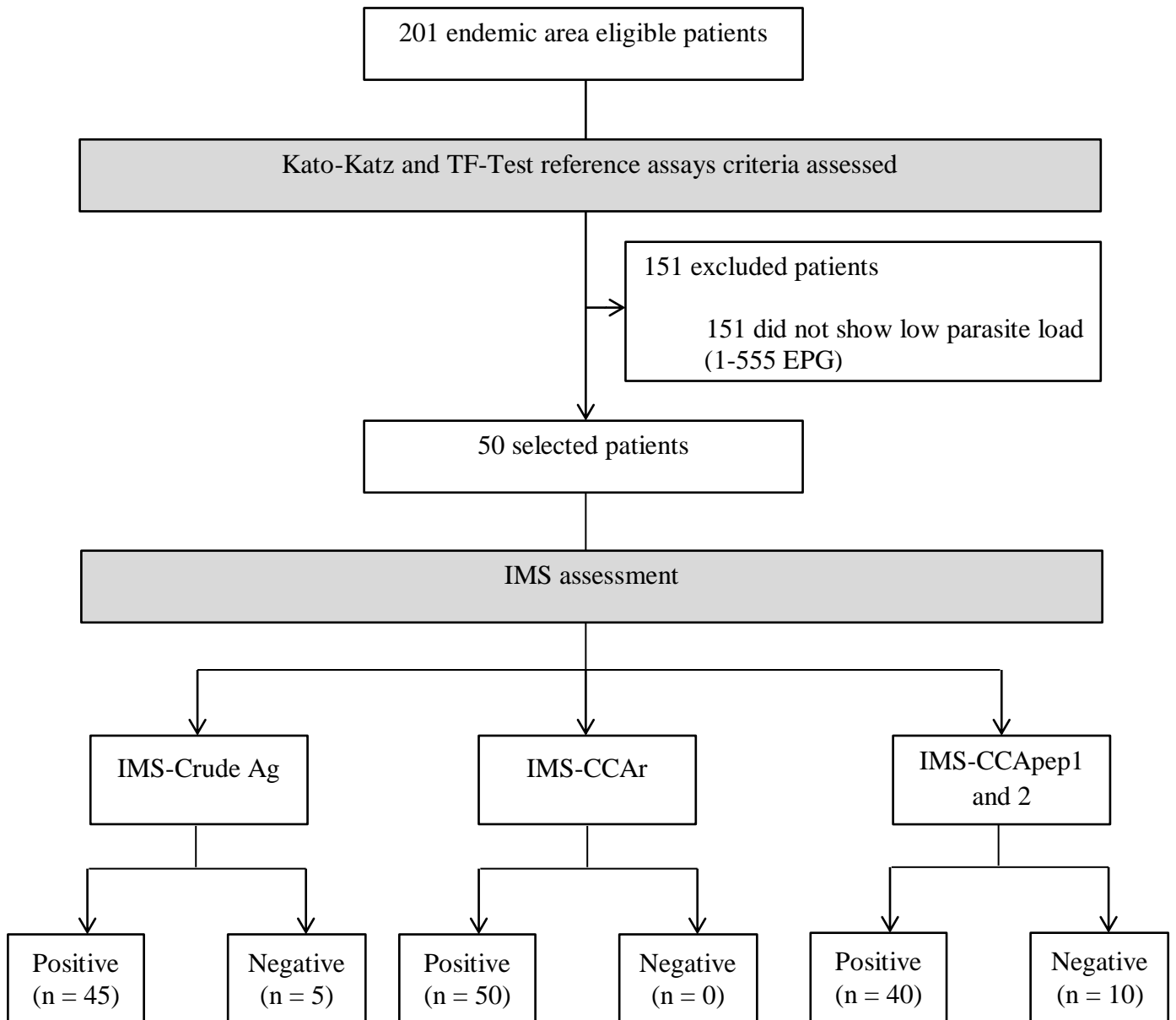

Supplement: Checklist S2 — Flowchart. Diagram that represents the sequencing of operations for the prospective study performed in the communities of Buriti Seco and Morro Grande in Pedra Preta, Brazil. (PDF) [file pntd.0002054.s002.pdf]
